# Adding colchicine to tocilizumab in hospitalized patients with severe COVID-19 pneumonia: An open-label randomized controlled trial

**DOI:** 10.1097/MD.0000000000030843

**Published:** 2022-09-30

**Authors:** Alaa Rahhal, Mostafa Najim, Amer Hussein Aljundi, Ahmed Mahfouz, Sumaya Mehdar Alyafei, Ahmed Awaisu, Mhd, Baraa Habib, Ibrahim Obeidat, Mohanad Mohammed Faisal, Meshaal Ali Alanzi, Arun Prabhakaran Nair, Areeg Elhassan, Abdullah Al-Dushain, Alaaeldin Abdelmajid Abdelmajid, Ahmed Elfadil Abdelgader, Ahmed Mahmoud Ahmed Moursi, Ahmad Eid Nazzal Alharafsheh, Mohd Ragheb Abou Kamar, Wael Goravey, Amr Salah Omar, Mohammed Abukhattab, Mohamad Yahya Khatib, Mohamed Gaafar Mohamedali, Muna A. Rahman AlMaslamani, Samar Alemadi

**Affiliations:** a Pharmacy Department, Hamad Medical Corporation, Doha, Qatar; b Internal Medicine Department, Rochester Regional Health - Unity Hospital, NY, USA; c Internal Medicine Department, Hamad Medical Corporation, Doha, Qatar; d College of Pharmacy, Qatar University, Doha, Qatar; e Infectious Diseases Department, Hamad Medical Corporation, Doha, Qatar; f Cardiothoracic Surgery/Cardiac Anaesthesia Department, Hamad Medical Corporation, Doha, Qatar; g Medical Intensive Care Department, Hamad Medical Corporation, Doha, Qatar; h Rheumatology Department, Hamad Medical Corporation, Doha, Qatar.

**Keywords:** colchicine, COVID-19, IL-6 antagonists, severe pneumonia, tocilizumab

## Abstract

Colchicine acts upstream in the cytokines cascade by inhibiting the nod-like receptor protein 3 (NLRP3) inflammasome while interleukin 6 (IL-6) receptor antagonists, such as tocilizumab, block the end result of the cytokines cascade. Hence, adding colchicine to tocilizumab with the aim of blocking the early and end products of the cytokines cascade, might reduce the risk of developing cytokine storm.

**Methods and analysis:**

We aim to conduct an open-label randomized controlled trial to evaluate the efficacy and safety of adding colchicine to tocilizumab among patients with severe COVID-19 pneumonia to reduce the rate of invasive mechanical ventilation and mortality. We will include patients with severe COVID-19 pneumonia who received tocilizumab according to our local guidelines. Enrolled patients will be then randomized in 1:1 to colchicine versus no colchicine. Patients will be followed up for 30 days. The primary outcome is the rate of invasive mechanical ventilation and will be determined using Cox proportional hazard model.

**Discussion:**

Given colchicine’s ease of use, low cost, good safety profile, and having different anti-inflammatory mechanism of action than other IL-6 blockade, colchicine might serve as a potential anti-inflammatory agent among patients with severe COVID-19 pneumonia. This study will provide valuable insights on the use of colchicine in severe COVID-19 when added to IL-6 antagonists.

**Ethics and dissemination:**

The Medical Research Center and Institutional Review Board at Hamad Medical Corporation in Qatar approved the study protocol (MRC-01-21-299). Results of the analysis will be submitted for publication in a peer-reviewed journal.

## 1. Introduction

The novel coronavirus infection (COVID-19) significantly contributes to increased mortality in many countries, with a continuously increasing number of infected cases worldwide.^[1,2]^ The leading cause of mortality due to COVID-19 is respiratory failure from acute respiratory distress syndrome (ARDS).^[3]^ The mechanisms by which COVID-19 induces ARDS is currently thought to be due to the activation of the innate immune system leading to hypercytokinemia and hence cytokine storm.^[4]^

COVID-19 disease course can be divided into three phases; early infection phase, where the virus penetrates host cells in the lung parenchyma; pulmonary phase, in which the viral propagation causes lung tissue injury and hence the host immune response is activated; and the inflammatory cascade, which is triggered by the viral RNA and it is comprised of inflammasome activation that drives the cytokine storm.^[4,5]^ SARS-CoV-2 acts on the macrophages to release the nod-like receptor protein 3 (NLRP3) inflammasome, leading to the conversion of pro-interleukin (IL)-1 and pro-IL-18 to their active forms.^[4,6]^ The production of IL-1 leads to interleukin 6 (IL-6) synthesis which is a cytokine that induces C reactive protein (CRP) and has been considered a major proinflammatory agent in the COVID-19 cytokine storm.^[7–10]^ Consequently, a few biological therapies that have been studied and used for the treatment of COVID-19 to target specific cytokines augmented by the disease, including anakinra for IL-1, and tocilizumab for IL-6.^[11]^ Early in the COVID-19 pandemic, the therapeutic efficacy of IL-6 antagonists was evaluated and showed conflicting results.^[12–15]^

Colchicine is one of the oldest anti-inflammatory therapies that is approved for treating and preventing acute gout attacks and familial Mediterranean fever.^[16,17]^ Colchicine has been shown to interfere with the activation of the NLRP3 inflammasome, reducing IL-1 production, which in turn prevents IL-6 release and neutrophils and macrophages activation.^[18–20]^ A few studies have evaluated the use of colchicine in hospitalized patients with COVID-19. The GRECO-19 trial was the first open-label randomized trial that evaluated the efficacy of colchicine versus usual care in non-critically ill hospitalized patients. The study included 105 patients and found a significant reduction in the primary outcome of a two-point deterioration on World Health Organization disease severity scale.^[21]^ Additionally, in a single center cohort study in Italy, the use of colchicine in 122 hospitalized patients with COVID-19 compared with 140 patients who received hydroxychloroquine and/or intravenous dexamethasone; and/or lopinavir/ritonavir, resulted in a significantly better survival rate at 21 days (84.2% vs 63.6% respectively, *P* = .001).^[22]^ The RECOVERY trial of colchicine in hospitalized COVID-19 patients found that colchicine was not associated with mortality or morbidity benefit in comparison to usual care.^[23]^ Moreover, there are a few ongoing trials in different countries evaluating the use of colchicine in COVID-19 in comparison to standard care, while excluding the concurrent use of IL-6 antagonists: (Italy [NCT04375202 and NCT04322565]; Spain [NCT04350320]; Argentina [NCT04328480]; Iran [NCT04360980]; USA [NCT04363437]).

Given the controversy around the therapeutic alternatives for COVID-19 pneumonia, adding colchicine to tocilizumab with the aim of blocking the early and end products of the cytokines cascade, might reduce the risk of developing cytokine storm. Therefore, we aim to conduct an open-label randomized controlled trial to evaluate the efficacy and safety of adding colchicine to tocilizumab among patients with severe COVID-19 pneumonia.

## 2. Methods and Analysis

### 2.1. Study design

This is a single center open-label randomized controlled trial aims to evaluate the efficacy and safety of using colchicine versus no colchicine among patients with severe COVID-19 pneumonia who received tocilizumab and admitted with severe COVID-19 pneumonia to COVID-19 tertiary facilities at Hamad Medical Corporation (HMC) in Qatar, including Hazm Mebaireek General Hospital (HMGH), Cuban Hospital (CH), and Communicable Disease Center (CDC). The study will cover the following objectives; primary: evaluate the efficacy of colchicine versus no colchicine in terms of the need of invasive mechanical ventilation among patients with severe COVID-19 pneumonia who received tocilizumab; secondary: assess the impact of colchicine on in-hospital mortality, duration of mechanical ventilation, and intensive care unit (ICU) length of stay among patients with severe COVID-19 pneumonia who received tocilizumab, evaluate the safety of using colchicine among patients with severe COVID-19 pneumonia who received tocilizumab.

### 2.2. Study population

The study will include patients admitted to HMC COVID-19 facilities, including HMGH, CH, and CDC with a confirmed diagnosis of severe COVID-19 pneumonia.

### 2.3. Inclusion criteria

Patients will be considered eligible to participate in the study, if they meet the following criteria:

1. Alert and conscious adults with the age of >18 years old
2. Severe COVID-19 pneumonia defined as SpO_2_ < 94% on room air at sea level, respiratory frequency > 30 breaths/minutes, or lung infiltrates >50%
3. Received tocilizumab within 10 days prior to enrollment
4. Tocilizumab is co-administered with a systemic corticosteroid
5. Agree to sign the written informed consent

### 2.4. Exclusion criteria

Patients will be ineligible to be recruited in case they have any of the following:

1. Severe COVID-19 pneumonia requiring invasive mechanical ventilation
2. Creatinine clearance calculated using Cockcroft-Gault equation <30 mL/min
3. End stage renal disease on hemodialysis
4. Alanine transaminase (ALT) and/or aspartate transaminase (AST) > 5 upper limit of normal
5. Pregnancy
6. Lactation
7. Concurrent use of a strong CYP3A4 inhibitor (e.g. clarithromycin, indinavir, itraconazole, ketoconazole, nefazodone, nelfinavir, ritonavir, saquinavir, telithromycin, atazanavir), a moderate CYP3A4 inhibitor (e.g. diltiazem, verapamil, fluconazole, amprenavir, aprepitant, fosamprenavir) or a P-gp Inhibitor (e.g. cyclosporine, ranolazine)

### 2.5. Randomization and treatment allocation

COVID-19 patients who meet the eligibility criteria of the study will be randomized using web-based randomization to receive colchicine (intervention arm) versus receiving no colchicine (control arm) in 1:1 ratio. Allocation numbers will be generated using the randomization website, and stratification will be balanced by gender (male and female) and age (<40 years, 40–60 years, >60 years), resulting in 6 strata.

### 2.6. Intervention

Participants allocated in the intervention arm will receive colchicine using the following dosing schedule:

1. Day 1: Colchicine 1 mg and 0.5 mg after 1 hour.
2. Day 2:
  • Weight >70 kg: 0.5 mg twice daily for 14 days, or discharge, whichever happens first
  • Weight <70 kg: 0.5 mg once daily for 14 days, or discharge, whichever happens first

In case of concomitant use of azithromycin:

1. Day 1: Colchicine 1mg
2. Day 2:
  • Weight >70 kg: 0.5 mg twice daily for 14 days, or discharge, whichever happens first
  • Weight < 70 kg: 0.5 mg once daily for 14 days, or discharge, whichever happens first

Throughout the 14-day course, in case of elevation of liver enzymes (ALT/AST) by more than 5 upper limit of normal and/or decrease in creatinine clearance to <30 mL/min, colchicine will be stopped, and outcomes will be censored.

### 2.7. Outcome measures

During the 30-day follow up period, the participants will be monitored for the following outcomes.

#### 2.7.1. Primary outcome

1. Rate of invasive mechanical ventilation

#### 2.7.2. Secondary outcomes

1. Time to invasive mechanical ventilation
2. Duration of invasive mechanical ventilation
3. 30-dayin-hospital mortality
4. ICU length of stay
5. Hospital length of stay
6. Adverse drug reaction (ADR) due to colchicine
7. Colchicine discontinuation rate

**Figure 1.**
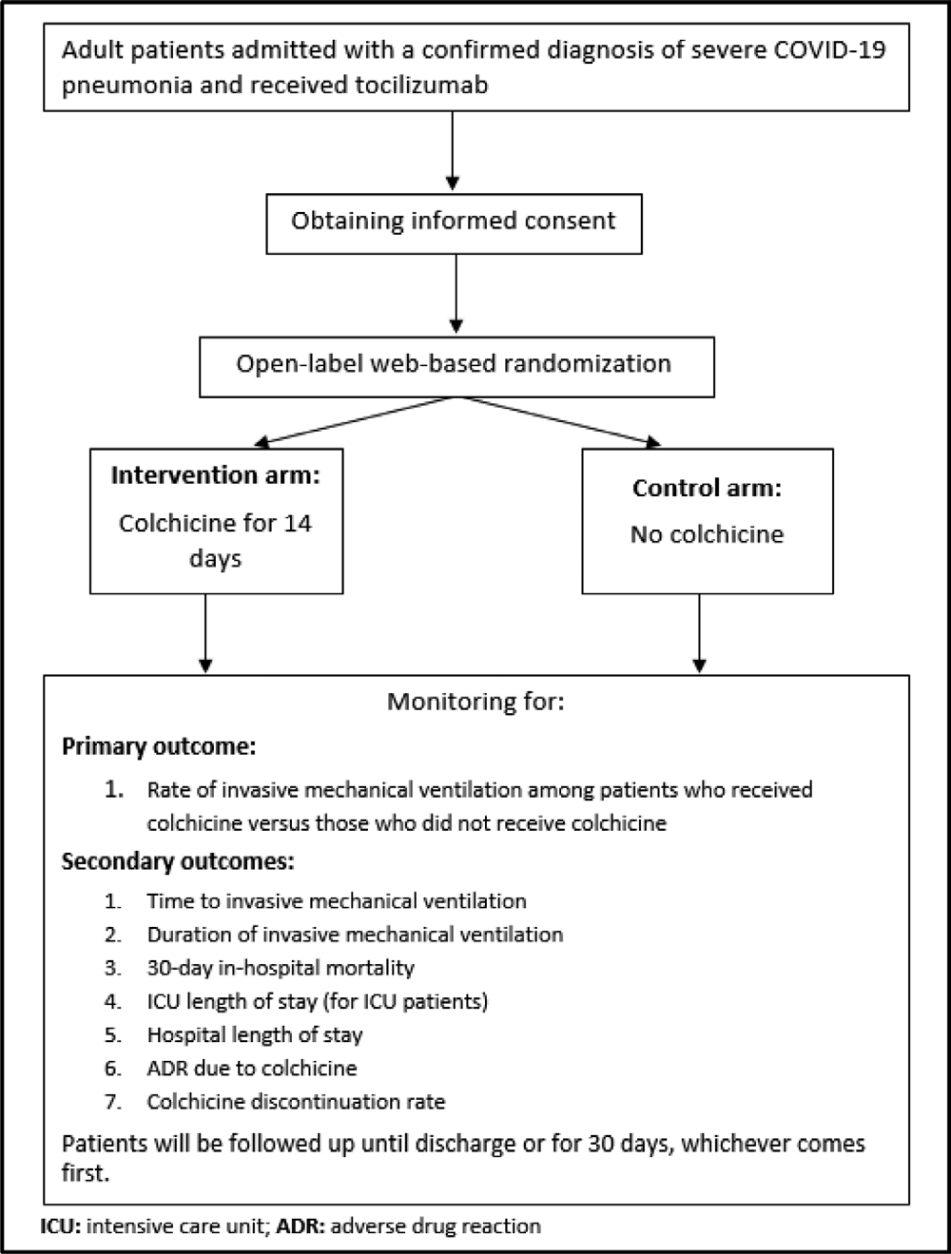
Trial design and flowchart.

Patients will be followed up until discharge or for 30 days, whichever comes first as shown in Figure 1.

### 2.8. Sample size

The rate of invasive mechanical ventilation was about 50% among COVID-19 patients in Qatar in 2020.^[24]^ Assuming the rate of invasive mechanical ventilation is about 50% in the control arm, a minimum sample size of 190 patients will provide 80% power to detect a relative risk reduction of approximately 40% and absolute risk reduction of 20% in the intervention group. To account for 20% dropout rate, the estimated sample size would be 230 patients randomized in 1:1 ratio (115: 115).

### 2.9. Statistical analysis

For the baseline characteristics, descriptive statistics in the form of mean and standard deviations or median and interquartile range will be calculated for interval variables and percentages for categorical variables. Chi-square or Fisher exact tests will be used for categorical variables to compare the two groups. Student t test will be applied for normally distributed variables and Mann–Whitney U test for non-normally distributed interval variables.

For the primary outcome of rate of invasive mechanical ventilation, it will be reported as percentages, and difference between the two arms will be evaluated using Chi-square or Fisher exact tests, as appropriate, and presented using absolute risk reduction and relative risk reduction. Additionally, Cox proportional hazard regression analysis (survival analysis) will be used to determine the association between colchicine use and time-to-primary outcome, with the adjustment of baseline imbalances between the two groups, if any. The results are to be presented as hazard ratio (HR), with a 95 % CI and *P* value <.05 will indicate statistical significance.

Secondary outcomes, including 30-day in-hospital mortality, ADR due to colchicine, and colchicine discontinuation rate will be reported as percentages, and difference between the two arms will be evaluated using Chi-square or Fisher exact tests, as appropriate, and presented using absolute risk reduction and relative risk reduction. Remaining outcomes, including time to invasive mechanical ventilation, duration of invasive mechanical ventilation, ICU length of stay, and hospital length of stay will be reported as continuous data in mean days or median days according to data distribution, and difference between the two arms will be evaluated using Student t test for normally distributed variables and Mann–Whitney U test for non-normally distributed variables. All reported p-values will be two-sided, and *P* value <.05 will indicate statistical significance. Statistical analyses will be performed using SPSS 24.0 statistical package.

### 2.10. Monitoring

#### 2.10.1. Safety monitoring

Colchicine use, like any other medication, can cause adverse drug events, and the most common adverse drug events reported with colchicine use are gastrointestinal: diarrhea, nausea, and vomiting.^[16]^

Other side effects that have been reported with colchicine use (with unknown frequency), include^[16]^:

1. Neurological: sensory motor neuropathy
2. Dermatological: alopecia, maculopapular rash, purpura, rash
3. Hematological: leukopenia, granulocytopenia, thrombocytopenia, pancytopenia, aplastic anemia
4. Hepatobiliary: elevated AST, elevated ALT
5. Musculoskeletal: myopathy, elevated creatine kinase, myotonia, muscle weakness, muscle pain, rhabdomyolysis
6. Reproductive: azoospermia, oligospermia

In view of strict safety precautions followed worldwide and in Qatar to limit the spread of COVD-19, investigators will not be able to assess the development of ADRs by direct contact with the patients. Therefore, the following steps will be followed:

1. After enrollment, investigators will seek safety information regarding the development of possible ADRs by contacting over the phone the managing teams of the enrolled patients during the follow-upperiod on daily basis.
2. Monitoring the enrolled participants for safety outcomes includes monitoring the development of the adverse drug events mentioned above, and if the study participant develops an adverse event due to colchicine, an adverse drug event form will be filled in the patient’s profile electronically within 24 hours of event, and it will be recorded in the data collection sheet.
3. The study principal investigator will be notified by the study investigator who identified the ADR to update the ADR report that will be reviewed by the data safety monitoring board (DSMB).
4. Severity of the ADR will be assessed using NCI Common Terminology Criteria for Adverse Events (CTCAE), v4.0.
5. Severe ADRs will reported by the principal investigator to the IRB.

Medical judgment will be used to determine the relationship, considering all relevant factors, including pattern of reaction, and the decision to stop colchicine will depend on the severity of the adverse reaction. In case of severe adverse events (Grade 3–5) due to colchicine, the medication will be stopped.

#### 2.10.2. Data safety monitoring board

The DSMB, an independent committee from the study investigators, will comprise 2 established clinical investigators, and 1 biostatistician who are independent from study investigators. The primary role of this board will be the continuous independent review of the study reports developed by the study principal investigator, concerning: study progress; effectiveness and safety reports, with the conduction of the formal interim analysis.

#### 2.10.3. Interim analysis

An interim analysis for efficacy and safety will be conducted after recruiting 50% of the panned sample size. The DSMB will employ the O’Brien-Fleming alpha spending function for stopping the study for efficacy and safety. The O’Brien-Fleming stopping rule will be used at an alpha of 0.005, and the *P* value for the final analysis will be conducted at .049. Cox regression will be used for the efficacy primary endpoint and Chi square test will be used for the safety endpoint (colchicine induced ADR). The stopping rule for futility will be based on conditional power. Discontinuation of the study because of futility will be considered at the time of the interim analysis if the accumulating data suggest a very low likelihood (<1%) of a statistically significantly lower primary end point event rate for colchicine arm.

## 3. Discussion

It remains controversial whether blocking IL-6 alone is effective. A meta-analysis of seven retrospective observational studies using tocilizumab in patients with severe COVID-19 found that tocilizumab resulted in lower all-cause mortality compared to placebo without achieving statistical significance. Furthermore, the risk of ICU admission and the need for mechanical ventilation were similar between the 2 groups.^[12]^ Later in 2021, the REMAP-CAP trial evaluated the use of a single injection of an IL-6 receptor blocker, tocilizumab or sarilumab, compared to placebo among 800 patients in need of respiratory or blood-pressure support or both and found that IL-6 antagonist resulted in an in-hospital mortality of 27%, as compared with 36% in the control group.^[13]^ The RECOVERY trial which included 4116 hospitalized patients with COVID-19 pneumonia demonstrated that tocilizumab significantly reduced all-cause mortality at 1 month in comparison to usual care (31% vs 35%, rate ratio 0·85; *P* = .0028).^[14]^ On the contrary, the COVACTA trial evaluated the use of tocilizumab versus placebo among 452 patients with severe COVID-19 pneumonia and it showed that the mortality rate was 19.7% in the tocilizumab group versus 19.4% in the control group.^[15]^

Colchicine, which is an old anti-inflammatory drug, targets multiple cytokines in the inflammatory cascade of the cytokine storm induced by COVID-19.^[16,17]^ This specifically makes it a potential anti-inflammatory therapy for COVID-19. Colchicine differs from IL-6 receptor blockers by having pleotropic mechanisms of action, acting upstream of the cascade of cytokines, and being an oral agent.

Given colchicine ease of use, low cost, good safety profile, having different anti-inflammatory mechanism of action than other IL-6 blockade, and the controversy of an effective therapy for COVID-19 so far, colchicine might serve as a potential anti-inflammatory agent among patients with severe COVID-19 pneumonia. Colchicine acts upstream in the cytokines cascade by inhibiting the NLRP3 inflammasome while IL-6 receptor antagonists block the end result of the cytokines cascade. The latter might be the reason behind the controversies surrounding tocilizumab efficacy in treating COVID-19 as it has been thought that the ongoing replication of the virus overwhelms the drug capacity to control the disease burden.^[25]^ Therefore, adding colchicine to IL-6 antagonists with the aim of blocking the early and end products of the cytokines cascade, might reduce the risk of developing cytokine storm and therefore the need for invasive mechanical ventilation and eventually death.

The main strength of our study is that it will evaluate the use of colchicine in COVID-19 pneumonia in a novel setting, where it will be used on top of tocilizumab, which is to the best of our knowledge is innovative in the literature of the pharmacological options for COVID-19.

To sum up, our study is an open-label randomized trial that will provide valuable insights on the use of colchicine in severe COVID-19 when added to IL-6 antagonists as it aims to evaluate the efficacy and safety of adding colchicine to tocilizumab among patients with severe COVID-19 pneumonia.

## 4. Ethics and Dissemination

This study will be carried out according to the research regulations of the Medical Research Center and Institutional Review Board at HMC in Qatar; it obtained the approval in August, 2021 (MRC-01-21-299). Results of the analysis will be submitted for publication in a peer-reviewed journal and international conferences.

## Author contributions

AR, AM, SMA, MAA, SA, and MN: conceived the study.

AR, and AA: designed the analysis plan

AR, MN, and AHA wrote the first draft of the protocol.

All authors reviewed and agreed on the final version of the manuscript.
